# Data Missing Not at Random in Mobile Health Research: Assessment of the Problem and a Case for Sensitivity Analyses

**DOI:** 10.2196/26749

**Published:** 2021-06-15

**Authors:** Simon B Goldberg, Daniel M Bolt, Richard J Davidson

**Affiliations:** 1 Department of Counseling Psychology University of Wisconsin - Madison Madison, WI United States; 2 Center for Healthy Minds University of Wisconsin - Madison Madison, WI United States; 3 Department of Educational Psychology University of Wisconsin - Madison Madison, WI United States; 4 Department of Psychology University of Wisconsin - Madison Madison, WI United States; 5 Department of Psychiatry University of Wisconsin - Madison Madison, WI United States

**Keywords:** missing data, randomized controlled trial, differential attrition, sensitivity analysis, statistical methodology, mobile phone

## Abstract

**Background:**

Missing data are common in mobile health (mHealth) research. There has been little systematic investigation of how missingness is handled statistically in mHealth randomized controlled trials (RCTs). Although some missing data patterns (ie, missing at random [MAR]) may be adequately addressed using modern missing data methods such as multiple imputation and maximum likelihood techniques, these methods do not address bias when data are missing not at random (MNAR). It is typically not possible to determine whether the missing data are MAR. However, higher attrition in active (ie, intervention) versus passive (ie, waitlist or no treatment) conditions in mHealth RCTs raise a strong likelihood of MNAR, such as if active participants who benefit less from the intervention are more likely to drop out.

**Objective:**

This study aims to systematically evaluate differential attrition and methods used for handling missingness in a sample of mHealth RCTs comparing active and passive control conditions. We also aim to illustrate a modern model-based sensitivity analysis and a simpler fixed-value replacement approach that can be used to evaluate the influence of MNAR.

**Methods:**

We reanalyzed attrition rates and predictors of differential attrition in a sample of 36 mHealth RCTs drawn from a recent meta-analysis of smartphone-based mental health interventions. We systematically evaluated the design features related to missingness and its handling. Data from a recent mHealth RCT were used to illustrate 2 sensitivity analysis approaches (pattern-mixture model and fixed-value replacement approach).

**Results:**

Attrition in active conditions was, on average, roughly twice that of passive controls. Differential attrition was higher in larger studies and was associated with the use of MAR-based multiple imputation or maximum likelihood methods. Half of the studies (18/36, 50%) used these modern missing data techniques. None of the 36 mHealth RCTs reviewed conducted a sensitivity analysis to evaluate the possible consequences of data MNAR. A pattern-mixture model and fixed-value replacement sensitivity analysis approaches were introduced. Results from a recent mHealth RCT were shown to be robust to missing data, reflecting worse outcomes in missing versus nonmissing scores in some but not all scenarios. A review of such scenarios helps to qualify the observations of significant treatment effects.

**Conclusions:**

MNAR data because of differential attrition are likely in mHealth RCTs using passive controls. Sensitivity analyses are recommended to allow researchers to assess the potential impact of MNAR on trial results.

## Introduction

### Background

In the world of mobile health (mHealth), high and rapid attrition is the rule rather than the exception [1]. This *law of attrition* [1] applies both to the use of mHealth interventions in naturalistic settings (eg, internet-based interventions for anxiety and depression [2]) as well as to studies designed to test the efficacy of mHealth interventions (eg, randomized controlled trials [RCTs] of smartphone-based interventions for mental health problems [3]). Attrition in naturalistic settings involves nonusage or discontinuation of usage, whereas attrition in research settings can involve these usage patterns along with dropouts from the study itself [4]. Nonusage and discontinuation of usage can limit the therapeutic potential of mHealth, and the development of methods to increase the sustained uptake of mHealth interventions is an area of active research [5,6]. In research contexts, attrition can not only attenuate therapeutic effects but can also produce additional problems such as reduced statistical power and the introduction of bias. This bias can skew results and limit the generalizability of the study findings (eg, only able to generalize to those who continue use). Various methods have been proposed to prevent attrition in mHealth research (eg, making interventions more engaging, implementing a *run-in* period before randomization, and including remainders and financial incentives [3,7]). However, to date, high attrition appears to be the rule rather than the exception of mHealth research [3].

Attrition in research contexts typically results in missing data. Some exceptions to this may include measures that continue to be assessed regardless of ongoing study participation (eg, smartphone app usage). Nonetheless, decades of methodological work have focused on characterizing the various types of missing data and developing statistical approaches for handling missingness [8,9] (for a more thorough discussion of the various types of missing data and methods for handling them, interested readers are directed to Enders [8] and Graham [9]; for a tutorial specifically geared to nonstatistician mHealth researchers, refer to Blankers et al [10]). There are three basic types of missing data that can be distinguished by their presumed cause as well as their impact on statistical tests [9]. The first and most benign type is data that are missing completely at random (MCAR). For example, an RCT testing a smartphone-based intervention for depression compared with a waitlist control group. In this context, it is common for posttreatment depression scores to be missing for a subset of participants [11]. If the missing data are MCAR, the cases with missing values can be viewed as a random sample of all cases. As such, the missing values did not systematically differ from the observed values. Statistical tests that ignore missing cases (eg, listwise deletion) provide unbiased estimates of parameter values, albeit with reduced statistical power. The second missing data type is data that are missing at random (MAR). For MAR data, the missing value (eg, posttest depression scores) does not depend on the missing value itself (ie, whether the missing score, if observed, would have been high or low) but depends on the observed data. For example, missingness may be more likely for those who had higher depression scores at baseline or were younger, but conditional upon such variables, the outcomes for missing cases resemble those of the observed cases (MCAR is actually a special case of MAR, specifically one in which the missing values are neither associated with observed values or missing values). Similar to MCAR, MAR data can also be analyzed in ways that produce unbiased parameter estimates, provided observed variables on which the missing value depends are included in the imputation and analysis model. Most recent advances in missing data analysis operate under the assumption of MAR. Multiple imputation (MI) and maximum likelihood (ML) are two widely used modern statistical methods that effectively use observed data for unbiased and statistically efficient (ie, not underpowered) analysis of MAR data and can also be applied to MCAR data.

Missing data that are MCAR and MAR are relatively straightforward to handle (so-called *ignorable* missing data [9]). In contrast, data that are missing not at random (MNAR) are a larger problem (ie, not ignorable), particularly when there is a substantial amount of missing data (eg, >5% [9]). For MNAR data, missingness depends on the value of unobserved data. For example, those who would have reported higher depression symptoms posttreatment may be more likely to drop out. The consequences of MNAR in RCTs can be substantial. In our depression RCT example, we found that participants in the active condition (ie, the intervention arm) were more likely to have missing posttest depression scores than the waitlist control (ie, passive control). It is generally impossible to demonstrate that data are MNAR, and simply having differential attrition does not necessarily indicate MNAR data or lead to biased results [12]. Although MNAR cannot be assessed directly, we might speculate that participants in the active condition who did not benefit as much from the smartphone-based intervention may be more likely to drop out of the study (refer to Crutzen et al [13] for similar possibilities offered to explain higher attrition in treatment vs control conditions in health behavior change interventions). This could be because of, for example, the greater effort required to participate in the treatment arm, especially if experiencing higher levels of depression. Such a relationship may well supersede what can be explained by the other measured variables for such subjects. In this case, the likelihood of having an unobserved posttest depression score is dependent on the value of the score itself, had it been observed. Thus, we are under the condition of MNAR. Further, the consequences of the MNAR on the estimation of treatment effects may be substantial, leading to an overestimation of the effect of the treatment because of missing observations. It is theoretically possible that the influence of MNAR data is reversed, with those dropping out experiencing better rather than worse outcomes (eg, dropping out of the study because one’s symptoms have already improved). This possibility is viewed as unlikely in related disciplines (eg, addiction research [14]). Lacking data or a strong rationale suggesting that missingness because of improved outcomes is likely in mHealth research, we focus on the more plausible MNAR mechanism of individuals who fail to respond to be those most likely to discontinue study participation.

Unlike MCAR and MAR missingness patterns, MNAR cannot be easily handled in a confident manner. Moreover, MNAR can have multiple causes, making it difficult to develop a single method that can universally address it, even in a single study. As a result, some form of sensitivity analysis is recommended to understand the possible effects of MNAR [12,15]. Advanced tools for evaluating the consequences of MNAR data have been developed, most notably selection models [16] and pattern-mixture models [17]. These models accommodate the joint distribution between the probability of missingness and the observed data and can be powerful techniques for evaluating the impact of MNAR. At once, understanding and implementing selection models or pattern-mixture models is a high bar for many applied researchers who may be faced with MNAR data. These models also involve untestable assumptions whose violations can produce biased results [8,18]. Thus, the application of MNAR procedures is undertaken more in the spirit of understanding the possible implications of missingness rather than explicitly correcting it. This approach is consistent with viewing missing data on a continuum from MAR to MNAR and focuses on evaluating whether the results are robust to MAR assumptions implicit in MI and ML analytic approaches [9].

Other methods have been proposed to assess the influence of MNAR data. The combination of high attrition and data that are potentially MNAR is not unique to mHealth research, and several approaches have come from the addiction field [19,20]. A classic example of MNAR data occurs in smoking cessation research, where individuals who drop out of the study are more likely to have returned to smoking. Historically, a widely used approach to handling missing smoking cessation data is simply to assume that missingness equals smoking [14]. This approach is considered conservative and is arguably preferred over treating the missing values as MAR or MCAR. However, assuming missing equals smoking can also introduce bias [21]; for example, if missingness is strongly related to group assignment in the context of an RCT and not all participants who drop out, in fact, return to smoking (eg, higher attrition in the waitlist vs nicotine patch condition).

Hedeker et al [14] offered a sensitivity analysis approach for evaluating the impact of MNAR on study results within the context of smoking cessation that could be adapted for use in mHealth research. Specifically, Hedeker et al [14] recommend evaluating the sensitivity of results to varying assumptions about the smoking status for those with missing data [14]. Models range, for example, from assuming a perfect association between missingness and smoking (ie, missing=smoking) to assuming that the odds of smoking for an individual with missing data are between 2 and 5 times higher than those with nonmissing data [14,22]. If the results are robust to variations in the assumed value of missing data, one can be more confident that the potential MNAR does not undermine the findings. If the results change, one can also characterize the point at which this occurs (eg, shifting from statistical significance to nonsignificance). Similar approaches have been proposed in other fields as well (eg, cost-effectiveness analyses) and incorporated into a broader MAR framework (eg, MI [23]).

Despite a longstanding acknowledgment that missing data are common in mHealth research [1], to our knowledge, there has not been a systematic investigation of the nature of missing data (ie, MAR vs MNAR) and no recent evaluation of the ways in which study authors are handling missing data (for an older review of missing data analysis techniques in internet-based interventions for anxiety and depression, refer to Christensen et al [2]). As noted, it is unfortunately not possible to definitively determine whether missing data are MAR or MNAR [8]; by definition, one cannot establish an association between the likelihood of a missing value and the unobserved value itself. Some readers may be familiar with Little [24] MCAR test, which is designed to evaluate the likelihood of MCAR across a data set. Although it is tempting to consider this as a reliable option for establishing missingness as MCAR, it has a number of substantial drawbacks, including low power (which can lead to failure to reject the null hypothesis that data are MCAR), unlikely and untestable assumptions (eg, shared covariance matrix among miss data patterns), and failing to identify specific variables that violate MCAR (ie, providing only an omnibus test [8]). In the absence of a method for determining whether data are MNAR, one could argue that it is incumbent upon researchers to consider whether their handling of potentially MNAR data yields biased results.

As noted above, a pattern of missingness that may be suggestive of MNAR in mHealth research is when missingness is higher in an active condition relative to a passive (eg, waitlist) control group. The context of an RCT is important for making this claim. Random assignment should produce groups balanced on all relevant covariates at baseline, including those that would predict drop out [25]. As attrition would be caused, at least in part, by group assignment (ie, active vs waitlist), it is, therefore, important to speculate on the primary mechanism by which treatment creates missingness. In the context of mHealth, one could easily imagine that active participants are more likely to drop out because of the increased burden associated with their intervention. Presumably, participants who find the burden of remaining engaged to exceed the benefits (or lack of benefits) they are experiencing may be most likely to drop out. Likewise, participants who experienced adverse reactions to the intervention itself would be more likely to drop out. In both instances—participants failing to realize benefits or experiencing adverse reactions—missing posttreatment data are likely MNAR, with unobserved scores on average reflecting less improvement than observed scores. Regardless of the specific cause, the meaning of missingness in the active condition will almost certainly not be equivalent to the missingness in waitlist control. This makes it problematic to treat missing data as reflecting the same outcomes as others in their respective groups, which is precisely what MAR methods do.

A recent meta-analysis of attrition in RCTs testing smartphone-based mental health interventions [3] found evidence consistent with this potential source of MNAR data. Linardon and Fuller-Tyszkiewicz [3] noted that active participants were significantly more likely to drop out of the RCTs than the passive control group participants (odds ratio [OR] 1.87, 95% CI 1.45-2.41, across all follow-up time points). In contrast, this differential attrition was not observed when an active control condition was used (OR 1.13, 95% CI 0.91-1.42). As Linardon and Fuller-Tyszkiewicz’s [3] study was not primarily focused on differential attrition, they did not further explore the possibility of MNAR or its implications, nor did they conduct standard meta-analytic sensitivity analyses for this specific effect (eg, trim-and-fill adjustment [26]). It would be valuable to extend this finding by systematically evaluating how differential attrition is handled statistically in these mHealth RCTs and examining study design features associated with higher rates of differential attrition (ie, meta-analytic moderators).

In addition to further clarifying how differential attrition and potential MNAR are handled within the mHealth literature, there is a need to understand the potential implications of MNAR for study outcomes. Selection models and pattern-mixture models are two promising approaches. Sensitivity analyses such as those recommended by Hedeker et al [14] for smoking cessation could also be readily adapted for mHealth research.

### This Study

This study has 2 primary aims. The first is to systematically review the analytic methods used to address missingness in a portion of the mHealth literature that has previously shown indications of potential MNAR. We examined 36 RCTs drawn from Linardon and Fuller-Tyszkiewicz’s [3] recent meta-analysis of smartphone-based mental health interventions that compared active interventions and passive controls. To examine statistical moderators of differential attrition, we coded attrition and study design features. We then cataloged how missing data were handled within these trials, focusing on whether the statistical approaches could handle MAR or MNAR data.

Our second aim is to present methods for evaluating the effects of MNAR that may be relevant to mHealth research. We illustrate the value of sensitivity analyses by applying an MI-based pattern-mixture model along the lines of Hedeker et al [14], as well as a simpler fixed-value replacement sensitivity analysis as examples of informative methods for evaluating the impact of MNAR. To illustrate these approaches, we use data drawn from a recent RCT of a smartphone-based mental health intervention comparing 2 active conditions with a waitlist control group [27].

## Methods

### Assessment of MNAR and Systematic Review of Missing Data Analytic Approaches

To evaluate study design features associated with differential attrition and the methods used to handle missing data, we reanalyzed and systematically reviewed RCTs included in the meta-analysis of attrition in smartphone-based mental health interventions by Linardon and Fuller-Tyszkiewicz [3]. This meta-analysis is recent and includes a reasonably large sample of RCTs (n=36 studies) that compared active treatment with a passive control condition (ie, waitlist or no treatment). We coded the completer and drop out sample sizes for both active and passive conditions at posttreatment to characterize differential attrition. These values were then converted to ORs using standard meta-analytic methods [26].

Log ORs and their variance were then aggregated using a random effects meta-analysis, weighted as typical using inverse variance [26] in the metafor R package (R Core Team). As ORs and the variance of ORs cannot be computed for cells with zeros, we conducted analyses using the Peto method [28], as recommended in the Cochrane handbook [29]. We also conducted analyses by adding a continuity correction for instances of empty cells (ie, 0.5 added to all cells in a study with an empty cell [30]). Heterogeneity of effect sizes was characterized using I^2^ (ie, proportion of effect size variance that occurs between studies) and interpreted based on Higgins et al [31]. We assessed the potential influence of outliers by conducting a *leave-one-out* analysis in the metafor package [32] and using the *find.outliers* function in R [33] that excludes effect sizes whose CI do not overlap with the omnibus effect size CI.

We systematically reviewed several features of the included studies. These included the overall sample size, overall dropout rate, whether potential differential attrition was statistically evaluated (ie, comparing dropout rates for active vs passive conditions), whether differential attrition was detected, the approach used for handling missing data, whether a modern MAR data analytic approach was used (ie, MI or ML), and whether a sensitivity analysis was conducted to evaluate the potential impact of MNAR data. To evaluate whether these study characteristics were linked with differential attrition, we tested them as moderators [26]. All analyses were conducted using R [34].

### MNAR Sensitivity Analysis

We used data from a recently conducted RCT testing a smartphone-based mental health intervention [27] to illustrate 2 sensitivity analysis approaches for MNAR data. As many mHealth RCTs include pre- and posttreatment assessments on a continuous variable, we apply these sensitivity analyses using data of this kind. In this study, 2 versions of an active smartphone-based meditation intervention were compared with a waitlist control on changes in psychological distress for 8 weeks (n=343). The original RCT included 3 time points (pretest, midtreatment, and posttreatment), and the primary models used multilevel modeling with ML estimation. However, in keeping with the possibility of MNAR data, attrition was higher in the active intervention than the waitlist (OR 2.10, 95% CI 1.34-3.33).

The first approach is a variant of the pattern-mixture model [23]. First, one conducts MI, imputing missing values based on available data (eg, pretest scores and demographics). Code in Multimedia Appendix 1 implements this in R using the jomo [35], mitools [36], and mice [37] packages with 100 multiply imputed data sets. It is worth noting that a limitation of MI in this context is the likely simulation of a positive treatment effect in the missing outcomes (assuming a positive treatment effect is seen in the observed outcomes), which may not be correct in the presence of MNAR. Thus, we next modify the imputed (ie, previously missing) posttest values using an *offset* parameter representing varying MNAR conditions. In our example, we assume progressively worse outcomes for those with missing posttest values. As a lower distress score is better, we add positive constants defined in relation to the residual SD from a model predicting posttest scores controlling for pretest scores and group status. As the added positive constant increases, the assumed outcome for missing observations becomes progressively worse. To aid in interpretability, we followed Cohen [38] effect size convention and added this value multiplied by 0.20, 0.50, 0.80, 1.10, and 1.40 to the multiply imputed values for cases with missingness. For example, the deviation applied for the 0.20 condition is as follows:

Missing = Multiply imputed value + 0.20 × SD_Model_**(1)**

A possible limitation of this approach is that, in the possible absence of useful covariates in predicting missing outcomes, all missing observations will be generated with large SDs, implying a high degree of uncertainty in the missing outcomes. Thus, even when introducing the offset parameter following the pattern-mixture strategy, the observed variability in the missing observations will still be large. To the extent that we should not confuse lack of knowledge about missing outcomes with actual variability in the missing outcomes, a sensitivity analysis that also considers a fixed-value replacement for missing observations can be useful. Therefore, we also applied a second sensitivity analysis approach outside the context of MI. Consistent with our strategy, this second approach focuses on estimating residualized change scores, although simple change scores could also be used. Once residualized change scores are imputed for missing cases, nonparametric tests (eg, Wilcoxon signed-rank test) can then be conducted using these values to compare changes in the active and passive conditions while avoiding statistical drawbacks associated with conducting parametric tests using single imputed data (eg, artificially deflating SE by treating imputed values as if they were observed values [8]). Similar to the approach described above, to evaluate the influence of potential MNAR data, we tested varying assumptions about the meaning of missing posttest data from complete case analysis to a worst-case scenario. The first analysis assumes that the missing data are MCAR and uses complete cases.

Complete case analysis: Missing=NA **(2)**

For the worst-case scenario, the missing data were assumed to reflect the worst possible observed outcome. Residualized change is operationalized as the observed posttreatment score minus the predicted posttreatment score based on pretreatment. For an outcome such as distress, in which lower values are preferred (ie, lower distress), a larger (ie, more positive) residual indicates a smaller decline in distress (for negative values), or even an increase in distress over time (for positive values). For an outcome in which higher scores were better (eg, well-being), one would simply reverse this approach (ie, replace missing values with the minimum observed residual). In our example, the worst-case scenario replaces the missing values with the maximum value of the observed residualized change scores:

Worst-case scenario: Missing=Maximum_Residual_**(3)**

We then evaluated possibilities between the complete and worst-case scenarios, with missing values imputed to be 0.20, 0.50, and 0.80 SD from the mean residualized change score. These specific values were chosen to reflect small, medium, and large deviations based on Cohen [38] guidelines. Again, as a lower score is better for distress, these deviations were added to the mean residualized change score (the mean residual is expected to be zero but is included here for the sake of completeness):

Small MNAR deviation: Missing = Mean_Residual_ + 0.20 × SD_Residual_**(4)**

Medium MNAR deviation: Missing = Mean_Residual_ + 0.50 × SD_Residual_**(5)**

Large MNAR deviation: Missing = Mean_Residual_ + 0.80 × SD_Residual_**(6)**

For example, psychological distress in the RCT by Goldberg et al [27] was a composite of 3 measures assessing depression, anxiety, and stress, which were combined into a single measure and scaled to z units (ie, mean 0, SD 1). The mean residualized change in psychological distress was 0 (SD 0.65), and the maximum residualized change in psychological distress was 2.3. Therefore, the worst-case scenario replaced all the missing residualized change scores of 2.3. In the midrange scenarios, missingness was replaced with a small deviation from the mean (0 + 0.2 × 0.65 = 0.13), a medium-sized deviation from the mean (0 + 0.50 × 0.65 = 0.33), and a large deviation from the mean (0 + 0.80 × 0.65 = 0.52). Wilcoxon signed-rank tests compared the rank sum for the active and passive conditions based on the complete case analysis and the 4 scenarios. All analyses were conducted using R [34]. Deidentified data [39] and the R code necessary for conducting the sensitivity analyses are included in Multimedia Appendix 1.

## Results

### Assessment of MNAR and Systematic Review of Missing Data Analytic Approaches

Linardon and Fuller-Tyszkiewicz’s [3] review included 36 RCTs that compared one or more active conditions with a waitlist control condition. Intention-to-treat and completer sample sizes, along with study characteristics related to missing data analysis, are included in Table 1. The average sample size per study, combined across active and passive conditions, was 143.53 (SD 118.66). Average attrition rates were numerically higher in the active condition (23.32%, SD 19.88%) than in the passive condition (15.36%, SD 15.51%), and 2 studies reported no attrition [40,41]. Among the 34 studies with attrition, a minority (11/34, 32%) statistically compared attrition rates between active and passive conditions. A total of 6 studies detected differential attrition, in all cases reporting higher attrition in the active conditions relative to the passive conditions.

**Table 1:** Attrition rates and study design characteristics.

Study	Tx^a^ ITT^b^	Tx drop	WL^c^ ITT	WL drop	Diff^d^	Method^e^	Multiple imputation	Maximum likelihood
Bakker et al [42]	234	146	78	25	N/A^f^	ANOVA^g^	Yes	No
Bidargaddi et al [5]	192	106	195	88	Yes, higher in active	*t* tests	Yes	No
Bostock et al [43]	128	5	110	4	N/A	ANOVA	No	No
Carissoli et al [40]	20	0	18	0	N/A	ANOVA	N/A	N/A
Champion et al [44]	38	9	36	3	No	MLM^h^	Yes	Yes
Enock et al [45]	206	38	36	0	N/A	MLM	No	Yes
Faurholt-Jepsen et al [46]	39	6	39	5	N/A	MLM	No	Unclear^i^
Hall et al [47]	76	34	25	13	N/A	MLM	No	Unclear
Horsch et al [48]	74	29	77	15	N/A	MLM	Yes	Unclear
Ivanova et al [49]	101	20	51	4	N/A	MLM	No	Yes
Kahn et al [50]	80	1	80	0	N/A	*t* tests	No	No
Krafft et al [51]	67	15	31	5	N/A	MLM	No	Yes
Kristjansdottir et al [52]	70	23	70	33	N/A	*t* tests	No	No
Kuhn et al [53]	62	11	58	6	No	ANOVA	Yes	No
Lee and Jung [54]	102	25	104	18	N/A	ANOVA	No	No
Levin et al [55]	12	0	11	0	N/A	MLM	No	Unclear
Levin et al [56]	59	13	28	5	No	MLM	No	Unclear
Lüdtke et al [11]	45	10	45	6	No	ANOVA	Yes	No
Lukas and Berking [57]	16	2	15	2	N/A	ANOVA	No	No
Ly et al [58]	36	3	37	2	N/A	MLM	No	Yes
Ly et al [41]	14	0	14	0	N/A	MLM	No	Yes
Marx [59]	46	2	50	0	N/A	ANOVA	No	No
Miner et al [60]	25	2	24	3	N/A	ANOVA	Yes	No
Moëll et al [61]	29	3	28	1	N/A	ANOVA	No	No
Oh et al [62]	39	1	20	4	N/A	ANOVA	No	No
Pham et al [63]	31	14	32	7	N/A	ANOVA	No	No
Proudfoot et al [64]	242	116	230	32	Yes, higher in active	MLM	Yes	Yes
Roepke et al [65]	190	152	93	57	Yes, higher in active	MLM	No	Yes
Rosen et al [66]	57	17	55	7	Yes, higher in active	MLM	No	Yes
Schlosser et al [67]	22	3	21	0	N/A	ANOVA	No	No
Stjernsward and Hansson [68]	196	60	202	42	N/A	ANOVA	Yes	No
Stolz et al [69]	60	18	30	7	No	MLM	Yes	yes
Tighe et al [70]	31	2	30	0	N/A	ANOVA	No	No
van Emmerik et al [71]	191	111	186	45	Yes, higher in active	MLM	Yes	Unclear
Versluis et al [72]	46	9	42	3	Yes, higher in active	MLM	No	Unclear
Yang et al [73]	45	3	43	4	N/A	ANOVA	No	No

^a^Tx: active treatment conditions.

^b^ITT: intention-to-treat sample size; drop=attrition at posttreatment assessment.

^c^WL: waitlist (or no treatment control condition).

^d^Whether differential attrition was tested and, if so, whether a between-group difference was detected.

^e^Primary data analysis method.

^f^N/A: not applicable (because of lack of missing data or differential attrition test not conducted).

^g^ANOVA: analysis of variance or related method (eg, analysis of covariance).

^h^MLM: multilevel model.

^i^Unclear whether multiple imputation estimator was used.

Most studies used multilevel modeling (17/36, 47%) or a variant of analysis of variance (eg, analysis of covariance, multivariate analysis of variance; 16/36, 44%) as the primary analytic approach, with 8% (3/36) of studies using a *t* test. Half (18/36, 50%) of the studies used ML or MI to handle missing data. MI was used in 31% (11/36) of studies, with multiple imputed data sets then analyzed using analysis of variance or *t* tests [5,42]. ML was used in combination with multilevel modeling in 28% (10/36) of the studies. An additional 19% (7/36) of studies used multilevel modeling but did not specify the estimator [72]. No study conducted a sensitivity analysis to evaluate the potential impact of MNAR data.

**Figure 1:**
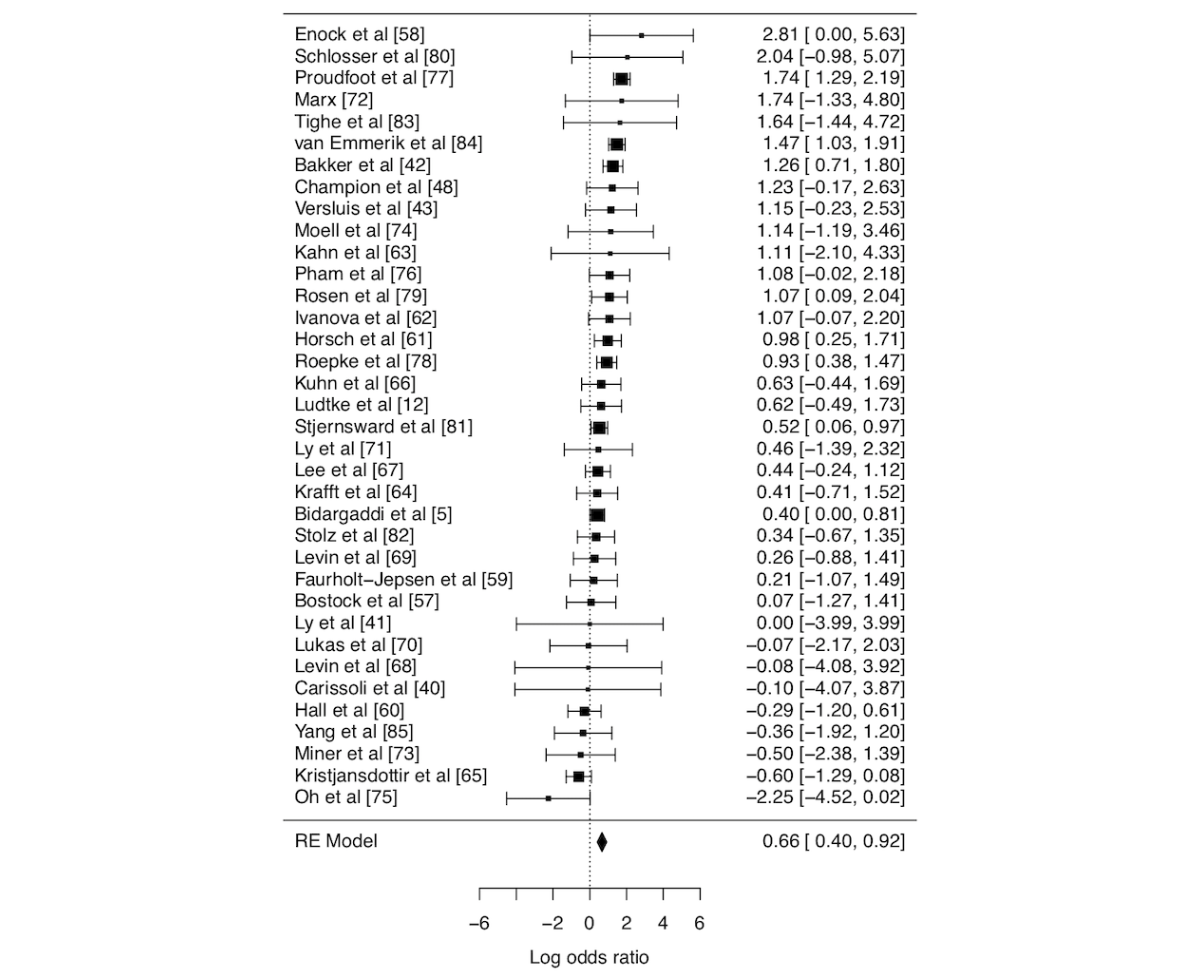
Forest plot displaying results of the meta-analysis. Effects sizes are in log-odds units, with larger values indicating higher attrition in active conditions relative to passive conditions. The size of points indicates relative weight in the meta-analysis (ie, inverse variance). RE: random effects.

Consistent with Linardon and Fuller-Tyszkiewicz [3], the results of our reanalysis provided a clear indication of differential attrition, with participants randomized to the active conditions approximately twice as likely to drop out relative to those in passive conditions (OR 1.94, 95% CI 1.50-2.51, using the Peto method; OR 2.22, 95% CI 1.93-2.54 using a continuity correction; both *P* values are <.001; Figure 1). The heterogeneity was moderate (I^2^=53.85%, 95% CI 19.46-71.09). The results were robust to *leave-one-out* analyses (OR range 1.82-2.10; all values of *P*<.001). The *find.outlier* function detected 4 outliers. Results were similar with these studies removed (OR 1.91, 95% CI 1.58-2.32; *P*<.001).

**Figure 2:**
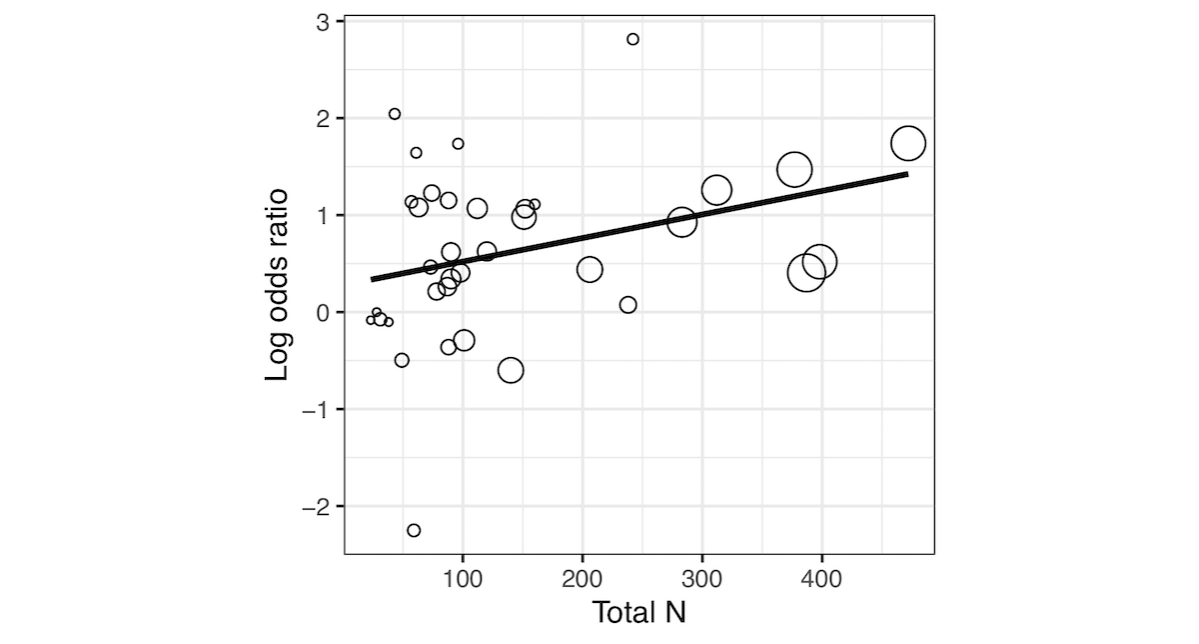
Results of meta-regression indicating that larger studies are associated with higher rates of differential attrition (ie, higher attrition in active vs passive conditions). Points are displayed relative to their weight in the meta-regression model (ie, inverse variance).

Several potential moderators were assessed using a meta-regression analysis. Active conditions were more likely to show higher attrition than passive conditions as the overall sample size increased (B=0.0022, 95% CI 0.0005-0.0039; note that all meta-regression coefficients are in log OR units; *P*=.01; Figure 2). Higher overall attrition was not associated with differential attrition (B=0.57, 95% CI −0.89 to 2.03; *P*=.45). Studies with higher differential attrition were marginally more likely to test for differences in attrition rates between active and passive conditions (B=0.49, 95% CI −0.002 to 0.99; *P*=.05, where testing=1 and not testing=0). There was no association between differential attrition rate and the likelihood of detecting differential attrition (B=0.55, 95% CI −0.19 to 1.29; *P*=.15, where detecting a difference=1 and not detecting a difference=0). The use of a modern missing data analysis method (ie, ML or MI) was associated with higher rates of differential attrition (B=0.73, 95% CI 0.25-1.2; *P*=.003, where use of ML or MI=1, no use of ML or MI=0).

### MNAR Sensitivity Analysis

Selection and pattern-mixture models are 2 valuable modeling strategies for handling MNAR (see Multimedia Appendix 2 [8,74,75] for a brief discussion of these methods and their limitations). New strategies and extensions of these approaches are continually being developed (eg, the index of local sensitivity to nonignorability [76]). However, many mHealth researchers may not be familiar with these methods. Selection models, in particular, require the missing data mechanism to be specified, which can be difficult to do. Moreover, a pattern-mixture approach of the kind described above often reflects a larger degree of uncertainty in the missing observations, an uncertainty that should not be confused with the presence of known variability in the missing outcome. Therefore, rather than abandon attempts to assess the potential impact of MNAR, mHealth researchers could consider approaches that simply make specific assumptions regarding anticipated outcomes for missing observations (ie, fixed-value replacement). By examining the estimated treatment effects in the presence of specific assumed outcomes for missing observations, we can similarly provide some insight into the degree to which varying missingness assumptions impact study results [14]. We illustrate both the pattern-mixture model and fixed-value replacement approaches using data drawn from the RCT by Goldberg et al [27].

Of the 343 participants randomized, 228 (66.5%) were assigned to 1 of the 2 active conditions, and 115 (33.5%) were assigned to the waitlist control. Consistent with the possibility of MNAR, noncompletion of posttreatment assessments was higher in the active condition (137/228, 60.1%) than in the waitlist condition (48/115, 41.7%; OR 2.10, 95% CI 1.34-3.33; *P*=.001). Goldberg et al [27] primary analyses used all 3 time points in multilevel models with ML estimation. The results indicated a steeper decline in psychological distress for the active conditions relative to the waitlist (time × group interaction; *P*<.001). Here, we examine how this result changes based on varying MNAR scenarios using either an MI-based pattern-mixture model approach [23] or a fixed-value replacement sensitivity analysis approach.

**Figure 3:**
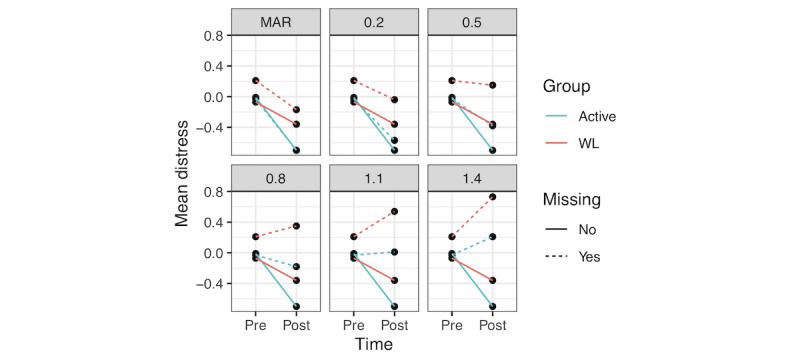
Pre- and posttreatment scores for active and passive conditions with varying constant offset parameters added to multiply imputed values for missing outcomes under conditions of missing not at random (ie, Missing). Values are in z-score units, scaled by distress at baseline (mean 0, SD 1). Panels illustrate trajectories with offsets ranging from 0.2 to 1.4 residual SD. The missing at random panel represents values derived using multiple imputation with no offset applied. MAR: missing at random; WL: waitlist.

Table 2 shows the estimates of the effect of group status on posttest distress, controlling for pretest distress across varying MNAR conditions within the pattern-mixture model framework. As the positive constant added (ie, offset parameter) increases, increasingly worse outcomes are assumed for the missing observations. Those in the active group continued to show larger declines in distress until imputed posttest distress scores were offset by a value of 1.10 or greater residual SD. Figure 3 depicts the impact of these varying MNAR conditions. The first panel displays the MAR-based estimates provided by MI, with imputed values closely following the trajectory of the respective groups. As MNAR conditions vary, the trajectories for imputed values become increasingly divergent from the observed scores, including the point that they reflect worsening scores with time.

**Table 2:** Results of pattern-mixture model sensitivity analysis based on multiple imputation^a^.

Model	Estimate^b^	*P* value
MAR^c^	−0.34	.002
0.20	−0.31	.004
0.50	−0.28	.01
0.80	−0.24	.03
1.10	−0.20	.08
1.40	−0.17	.16

^a^Models are based on varying assumptions regarding the meaning of missingness. Multiply imputed posttest values based on 100 imputations are offset [23] by varying amounts (ie, 0.20, 0.50, and residual SD).

^b^Coefficient for active group status (vs waitlist) predicting posttest distress scores controlling for pretest distress scores pooled across imputed data sets.

^c^MAR: missing at random (with no offset applied to posttest values).

**Figure 4:**
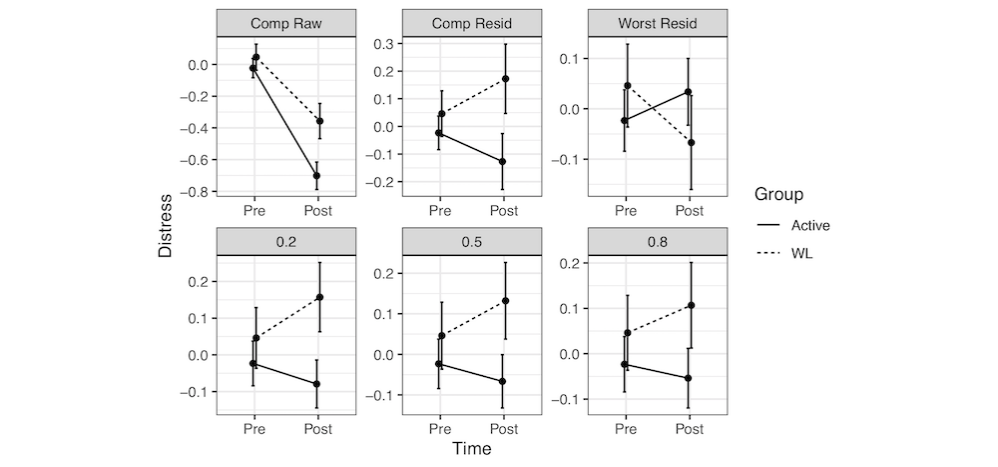
Pre- and posttreatment scores for active and passive conditions under varying missing not at random conditions using fixed-value replacement of missings. Pretreatment values represent z-scaled distress at baseline (mean 0, SD 1). Posttreatment values vary across plots. For Comp Raw, posttreatment values are posttreatment distress scaled based on baseline distress. Subsequent plots display residualized change scores z-transformed at posttreatment to aid in visual interpretation of relative, between-group pre-post change. Comp Resid computed posttreatment as baseline plus residualized change scores for completers only. Worst Resid replaced missing posttreatment Comp Resid values with the lowest improvement in distress. Subsequent figures (0.2, 0.5, 0.8) replaced missing posttreatment Comp Resid values with values 0.2, 0.5, and 0.8 SD worse than the mean residual. Comp: completer; Resid: residualized change; WL: waitlist.

We now turn to the results of the fixed-value replacement sensitivity analysis. Figure 4 visually depicts the impact of MNAR conditions on the trajectories of pre-post change for the active and passive groups using this approach. The first 2 panels (Comp Raw and Comp Resid) display changes for completers only (in raw units and residualized change units, respectively). However, if MAR is violated in the way hypothesized above, one would expect the trajectory for unobserved active group participants to be worse than the observed active group scores (ie, following a trajectory more similar to the passive condition). If the missing data are consistent with MAR, this adjusted trajectory can be adequately recaptured with observed data (eg, baseline variables), allowing unbiased estimation using ML and MI. However, in the case of MNAR, the likelihood of missingness depends upon the unobserved value itself, making it impossible to recapture from available data alone. The subsequent panels (Worst Resid, 0.20, 0.50, and 0.80) display the impact of varying assumptions about the meaning of missing values. As can be seen in the Worst Resid panel, assuming the worst observed outcome for those with missing data reverses the direction of effect, with control group participants now showing more improvement than active participants. One can see how the gap in outcomes between active and passive condition participants narrows as increasingly strong assumptions are made regarding the degree to which missing values deviate from observed values. As missingness was more prevalent in the active conditions, these modifications exerted a stronger influence on the change in the active group.

**Figure 5:**
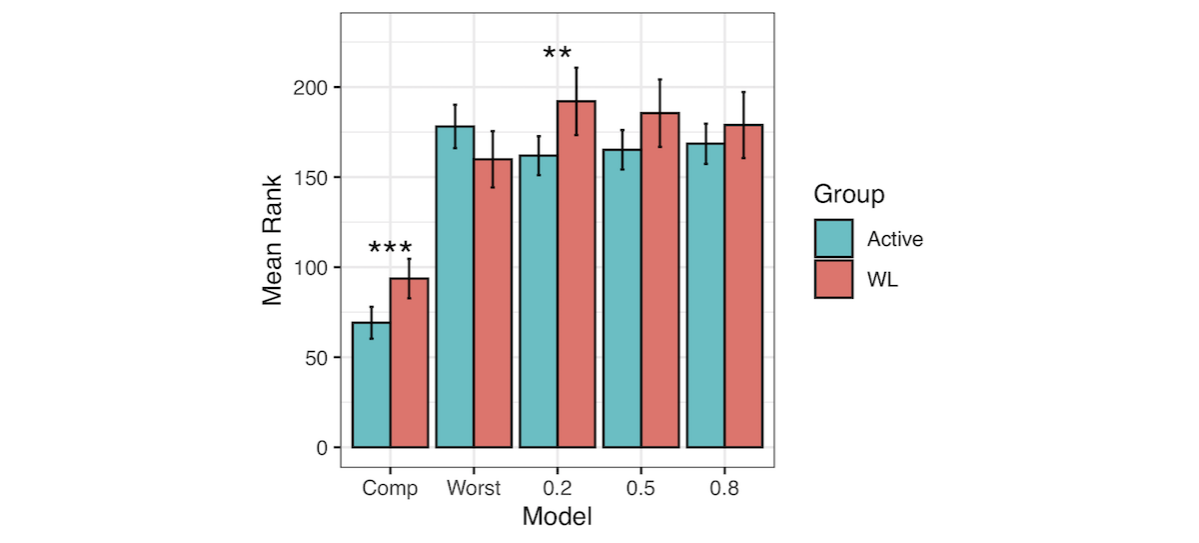
Results of Wilcoxon signed-rank test using a fixed-value replacement sensitivity analysis across varying missing not at random conditions. A lower mean rank indicates larger relative decreases in distress. Comp: completer sample; Worst: worst-case scenario which assumed missing values are equivalent to the worst outcome (ie, the smallest change in distress); 0.2, 0.5, 0.8: missing values assumed to be 0.2, 0.5, or 0.8 SD worse than the mean residualized change score; error bars: 1.96×SE; WL: waitlist. *P<.05, **P<.01, ***P<.001.

For null hypothesis testing purposes, we used nonparametric tests of mean residualized change scores. Consistent with the multilevel modeling results [27], the Wilcoxon signed-rank test favored the active conditions in the completer sample (mean ranks 69.11, 93.61, SD 43.01 and 45.90, for active and passive conditions, respectively, *P*<.001, where a lower rank indicates a larger decline in distress; Table 3). In the worst-case scenario, the direction of the mean rank difference flipped, now favoring the passive condition, although only marginally significantly (*P*=.08). Mirroring Figure 4, the influence of the varying missingness assumptions is apparent in Figure 5. The gap between active and passive conditions narrows, as missing data are assumed to reflect poorer and poorer outcomes. The pattern specifically indicates that statistical significance persists when missing values are assumed to be 0.20 above the mean residual but not 0.50 or higher. This result differs slightly from Goldberg et al [27], who detected statistical significance at an offset of 0.50. The discrepancy is because of Goldberg et al [27] calculating the SD for the residual without the group variable in the model. We recommend the inclusion of the group variable in the model, as the resultant SD is presumably more conservative, based on the assumption that an intervention increases the SD. Therefore, these results can be interpreted as robust to MNAR missing, in which the unobserved values deviate from the observed values only to a small degree, but not when showing moderate or larger deviations.

**Table 3:** Results of fixed-value replacement sensitivity analysis.

Group and model		Sample size, n (%)	Mean rank (SD)	SE	*P* value^a^
**Active**					
	Comp^b^	91 (39.9)	69.11 (43.01)	4.51	<.001
	Worst^c^	228 (100)	178.1 (93.05)	6.16	.08
	0.20^d^	228 (100)	161.89 (83.25)	5.51	.004
	0.50^d^	228 (100)	165.19 (84.25)	5.58	.05
	0.80^d^	228 (100)	168.52 (86.01)	5.70	.32
**Waitlist**					
	Comp	67 (58.3)	93.61 (45.9)	5.61	N/A^e^
	Worst	115 (100)	159.9 (85.62)	7.98	N/A
	0.20^d^	115 (100)	192.05 (102.27)	9.54	N/A
	0.50^d^	115 (100)	185.5 (102.26)	9.54	N/A
	0.80^d^	115 (100)	178.9 (100.34)	9.36	N/A

^a^*P* value from Wilcoxon signed-rank test comparing active and passive conditions across varying missingness assumptions.

^b^Comp: completer sample.

^c^Worst: worst-case scenario, which assumed missing values are equivalent to the worst outcome (ie, smallest change in distress).

^d^0.20, 0.50, 0.80: missing values assumed to be 0.20, 0.50, or 0.80 SDs worse than the mean residualized change score.

^e^N/A: not applicable.

## Discussion

### Principal Findings

This study had two primary aims: to systematically evaluate the handling of a potential source of MNAR data in mHealth research—differential attrition—and advocate for sensitivity analyses as a family of strategies that might be used to assess the impact of MNAR data. At the broadest level, results suggest that MNAR data are likely to be a problem in mHealth research and one that, to date, has not been adequately addressed. As reported by Linardon and Fuller-Tyszkiewicz [3], the substantially higher attrition in active relative to passive conditions in RCTs testing smartphone-based mental health interventions is marked; active participants were approximately twice as likely to drop out of the study. Although it is impossible to say what the missing posttreatment data would have shown had it been collected, it is plausible that those dropping out from the active conditions were less likely to have benefited from the mHealth intervention (or at least that the benefits they were experiencing did not outweigh the costs of remaining in the study). Thus, the observed values may overestimate treatment effects for those under active conditions. Given that the likelihood of missingness is related to the unobserved values themselves, these data would be MNAR.

Although patterns of attrition consistent with potential MNAR data were detected in the literature as a whole, only a minority of the included studies tested for differential attrition. However, in keeping with the literature-wide pattern of differential attrition, 6 of the 11 studies comparing attrition rates between active and passive conditions detected higher attrition in the active conditions, whereas the remaining 5 studies failed to detect a difference. Despite the possibility of MNAR data, none of the 36 studies directly assessed the potential influence of MNAR data on the study results. Half of the studies employed other modern missing data methods, such as MI or ML. These approaches have many strengths and are certainly preferred over historical approaches for handling missingness (eg, last observation carried forward and complete case analysis [9]). Encouragingly, it appears that less sophisticated missing data analysis techniques (eg, last observation carried forward [2]) are being replaced by modern methods. However, both MI and ML rely on the assumption that data are MAR; therefore, missing values can be reliably determined based on measured variables. Importantly, they are not robust to MNAR [8].

Moderator analyses further characterized the correlates of differential attrition. The results indicated that differential attrition was more likely to occur in larger studies. Unfortunately, this association could produce a pernicious source of bias within the literature, as larger studies are presumably the ones most looked to when evaluating evidence of efficacy and are likely to carry more weight in meta-analyses examining efficacy. Interestingly, studies with higher differential attrition were only marginally more likely to assess differential attrition. It may be that differential attrition is simply not recognized or acknowledged as a potential concern worth assessing, even when dropout rates differ. Somewhat counterintuitively, studies with higher differential attrition were not more likely to detect differential attrition when assessed. This lack of association could be because of the limited statistical power for the moderator test itself [77], as only 11 studies tested for differential attrition. Statistical power may also be low in primary studies themselves. For example, Champion et al [44] did not detect differential attrition in their sample of 74 participants, although active participants were 3.42 times more likely to drop out of the active condition relative to the passive condition. It appears that researchers are more likely to use modern missing data analysis techniques (ML/MI) when differential attrition is higher, which is preferred to techniques that are not robust to even MAR data (eg, complete case analysis and last observation carried forward). Nonetheless, these techniques are not capable of eliminating the bias associated with MNAR data.

Perhaps the most notable finding of our review is that none of the included studies conducted a sensitivity analysis to evaluate the potential influence of MNAR data on study findings. Although several meta-analyses suggest that smartphone-based mental health interventions produce benefits relative to waitlist control conditions [78-80], the lack of sensitivity analyses coupled with literature-wide differential attrition makes the apparent efficacy more tenuous.

The primary aim of this study is to encourage mHealth researchers to consider sensitivity analyses to assess the potential impact of MNAR missingness, particularly when differential attrition is present. Several modern techniques exist for evaluating the potential impact of MNAR missingness, including selection models and pattern-mixture models that have been discussed. As most of these methods have limitations (eg, they are heavily influenced by untestable assumptions) and may not be within the current analytic repertoire of many mHealth clinical trialists, we presented an MI-based pattern-mixture model sensitivity analysis approach adapted from smoking cessation research [14,22] as well as a fixed-value replacement sensitivity analysis approach as examples of more user-friendly strategies for evaluating the impact of MNAR data. These methods are fairly straightforward to implement using a continuous outcome variable assessed at pre- and posttreatment—a typical situation for mHealth research [80,81]—and move beyond the traditional MAR methods currently emphasized in mHealth research. An attractive feature of these sensitivity analyses is that one can visually and statistically evaluate the impact of varying missingness assumptions on the pattern of findings. As these assumptions would only apply in cases of missing data, they would have a minimal impact on the results when missingness is low (eg, <5% [9]). As expected, the actual impact of varying MNAR assumptions will be sensitive to other patterns in the data (eg, trajectories of change for waitlist control participants because of regression to the mean or natural history). Thus, they do not imply a particular direction of influence but rather evaluate a range of possible impacts based on deviations from the observed data.

It is worth noting that the 2 sensitivity analysis approaches illustrated in this study provided somewhat discrepant conclusions regarding the degree to which data from Goldberg et al [27] were robust to MNAR conditions. This fact highlights the value of sensitivity analyses and the importance of authors using various approaches and assumptions to evaluate the strength of their findings. These differences are also illuminating. In particular, the MI-based pattern-mixture model approach suggested that the results were robust to MNAR deviations that were large (ie, 0.80) but not larger, whereas the simpler sensitivity analysis approach indicated that the results were not robust above small deviations (ie, 0.20). Figure 3 illustrates a plausible explanation for this: the MI-based approach makes the initial assumption that missing values are similar to observed values unique to each group. Thus, the fact that the active group improved overall produced improvement in the imputed change for missing active participants. In contrast, the fixed-value replacement approach did not adjust the expected residualized change scores based on the group status. We contend that both approaches may provide a valuable perspective on MNAR sensitivity and should simply be interpreted in light of their underlying assumptions.

### Limitations and Future Directions

This study had several important limitations. The first and broadest limitation is that we cannot definitively conclude that the observed differential attrition necessarily results in MNAR data. It is possible that remaining in the study was because of factors unrelated to changes in study endpoints (ie, distress). Likewise, drop outs in the active group could have been because of participants not using the smartphone app and losing interest in the study because their psychological symptoms had already improved (as can be the case in psychotherapy [82]), which could produce an MNAR bias in the opposite direction (ie, missing values are better, not worse). As is typical for research on missing data, the data necessary to test for MNAR are by definition missing. The methods proposed here could certainly be extended to evaluate potential *best-case scenarios*, in which missing observations reflect better rather than worse outcomes or when missingness has different meanings depending on group assignment (eg, worse outcomes for active conditions but better outcomes for passive condition). Second, we only evaluated the degree and correlates of differential attrition in a small subset of the large and rapidly growing mHealth literature. It is possible that researchers are improving their ability to retain study participants and adherence strategies being investigated [6,83] may be decreasing attrition in the active conditions. Future reviews may see less evidence of this potential source of MNAR data. Similarly, there are mHealth RCTs that conducted sensitivity analyses to evaluate MNAR (eg, pattern-mixture models [84]), even though none of the 36 RCTs with passive controls we evaluated did so. Third, we focused only on differential attrition in smartphone-based RCTs. It is conceivable that higher attrition in active than passive conditions is somehow idiosyncratic to this delivery platform. An important future direction would be to evaluate differential attrition in other mHealth delivery formats (eg, internet-based interventions). Fourth, we explored only a few examples of possible methods for addressing sensitivity to MNAR data. Nonetheless, we hope our introduction of these approaches with corresponding R syntax encourages mHealth researchers to begin implementing and perhaps even testing and developing strategies for addressing the missing data realities of mHealth.

Several future directions follow naturally from this study. MNAR sensitivity analyses could be integrated into future mHealth RCTs. For instances with longitudinal data, more complex pattern-mixture models may be especially attractive [84]. For studies with fewer time points, approaches such as those introduced here may be worthwhile. If a specific sensitivity analysis approach were to become widely used, it could provide researchers with a common metric for evaluating the potential influence of differential attrition as a source of MNAR on study results. An approach based on readily interpretable metrics (eg, Cohen *d*) could be helpful, although there are certainly many viable possibilities, many of which may have advantages over the strategy introduced here. This is an area of active research, and new and much more sophisticated methods are regularly becoming available [76].

Short of incorporating sensitivity analyses into mHealth RCTs, researchers could, at a minimum, test for differential attrition, especially when comparing active and passive conditions. Acknowledging the potential influence of MNAR, when differential attrition is present, can allow readers to more accurately evaluate study findings in light of this limitation. A way to assess the potential impact of MNAR because of differential attrition would be through reanalysis of published mHealth RCTs, especially large trials that were seen to have higher rates of differential attrition. Reanalyses of this kind could help determine the degree to which findings are sensitive to varying MNAR assumptions, and by extension, the degree to which conclusions drawn from the broader literature may be similarly influenced. Another future direction is intentionally adopting methods that decrease attrition generally [85], given that differential attrition and associated MNAR data are less concerning when the amount of missing data is small. Finally, it could be valuable to investigate differential attrition for in-person interventions as well. To our knowledge, no such meta-analysis exists, although the same potential risk of bias because of MNAR may be applied.

### Conclusions

Attrition is a persistent thorn in the side of mHealth clinical trialists [1]. Modern missing data methods such as MI and ML successfully minimize the negative impact of some types of missing data (MCAR and MAR), restoring statistical power and reducing bias in parameter estimates. However, these methods cannot remove the bias associated with MNAR data.

Evidence of differential attrition supports the possibility that MNAR may be a common problem in mHealth RCTs with passive controls and one that is largely unacknowledged to date. Sensitivity analyses offer an approach for establishing the impact of differential attrition on the study results.

## Appendix

Multimedia Appendix 1 R code for conducting sensitivity analysis.

Multimedia Appendix 2 Model-based approaches for handling missing not at random data.

## Abbreviations

MAR: missing at random
MCAR: missing completely at random
mHealth: mobile health
MI: multiple imputation
ML: maximum likelihood
MNAR: missing not at random
OR: odds ratio
RCT: randomized controlled trial
