# Supplementary material for: Data Missing Not at Random in Mobile Health Research: Assessment of the Problem and a Case for Sensitivity Analyses
Source: J Med Internet Res. 2021 Jun 15;23(6):e26749. doi: 10.2196/26749 (PMC8277392; doi:10.2196/26749)
Supplement: Multimedia Appendix 1 [file jmir_v23i6e26749_app1.docx]

Multimedia Appendix 1. R code for conducting sensitivity analysis

#Pattern mixture model-based approach

df.rct$t3.miss <- ifelse(is.na(df.rct$t3),yes=1,no=0)

resid.sd <- sd(resid(lm(t3 ~ t1 + active, df.rct)))

#MI implementation

#Multiple imputation version

library(jomo);library(mitools);library(mice)

set.seed(1234)

imp100<-jomo1(dplyr::select(df.rct,-t3.miss),

nimp=100)

outjomo<-subset(imp100,Imputation>0)

mi_list <- imputationList(split(outjomo, outjomo$Imputation))

mi_results <- with(mi_list, lm(t3 ~ t1 + active))

summary(pool(as.mira(mi_results)))

round(summary(pool(as.mira(mi_results)))[,-1],3)

df.rct$id <- 1:dim(df.rct)[1]

outjomo <- merge(outjomo,df.rct[,c("id","t3.miss")])

outjomo <- arrange(outjomo,Imputation,id)

#Adding constant for varying MNAR assumptions

outjomo <- outjomo %>%

mutate(

t3.2 = t3,

t3.5 = t3,

t3.8 = t3,

t3.110 = t3,

t3.140 = t3

)

outjomo <- outjomo %>%

mutate(

t3.2 = t3 + t3.miss*resid.sd*.2,

t3.5 = t3 + t3.miss*resid.sd*.5,

t3.8 = t3 + t3.miss*resid.sd*.8,

t3.110 = t3 + t3.miss*resid.sd*1.1,

t3.140 = t3 + t3.miss*resid.sd*1.4,

)

#now models

df.out <- data.frame(model = c("MAR","0.20","0.50","0.80","1.10","1.40"),

est = NA, p = NA)

mi_list <- imputationList(split(outjomo, outjomo$Imputation))

mi_results <- with(mi_list, lm(t3 ~ t1 + active))

out <- summary(pool(as.mira(mi_results)))

df.out[df.out$model=="MAR","est"] <- round(out$estimate[3],2)

df.out[df.out$model=="MAR","p"] <- round(out$p.value[3],3)

mi_list <- imputationList(split(outjomo, outjomo$Imputation))

mi_results <- with(mi_list, lm(t3.2 ~ t1 + active))

out <- summary(pool(as.mira(mi_results)))

df.out[df.out$model=="0.20","est"] <- round(out$estimate[3],2)

df.out[df.out$model=="0.20","p"] <- round(out$p.value[3],3)

mi_results <- with(mi_list, lm(t3.5 ~ t1 + active))

out <- summary(pool(as.mira(mi_results)))

df.out[df.out$model=="0.50","est"] <- round(out$estimate[3],2)

df.out[df.out$model=="0.50","p"] <- round(out$p.value[3],3)

mi_results <- with(mi_list, lm(t3.8 ~ t1 + active))

out <- summary(pool(as.mira(mi_results)))

df.out[df.out$model=="0.80","est"] <- round(out$estimate[3],2)

df.out[df.out$model=="0.80","p"] <- round(out$p.value[3],3)

mi_results <- with(mi_list, lm(t3.110 ~ t1 + active))

out <- summary(pool(as.mira(mi_results)))

df.out[df.out$model=="1.10","est"] <- round(out$estimate[3],2)

df.out[df.out$model=="1.10","p"] <- round(out$p.value[3],3)

mi_results <- with(mi_list, lm(t3.140 ~ t1 + active))

out <- summary(pool(as.mira(mi_results)))

df.out[df.out$model=="1.40","est"] <- round(out$estimate[3],2)

df.out[df.out$model=="1.40","p"] <- round(out$p.value[3],3)

#Now figures

df.sum <- outjomo %>%

group_by(id) %>%

summarize(

active = mean(active),

t1 = mean(t1),

t3 = mean(t3),

t3.miss = mean(t3.miss),

t1.2 = mean(t1),

t1.5 = mean(t1),

t1.8 = mean(t1),

t1.110 = mean(t1),

t1.140 = mean(t1),

t3.2 = mean(t3.2),

t3.5 = mean(t3.5),

t3.8 = mean(t3.8),

t3.110 = mean(t3.110),

t3.140 = mean(t3.140)

)

head(df.sum)

df.sum$active.t3.miss <- paste(df.sum$active,df.sum$t3.miss,sep="_")

vars <- c("t1","t1.2","t1.5","t1.8","t1.110","t1.140",

"t3","t3.2","t3.5","t3.8","t3.110","t3.140")

out <- psych::describeBy(df.sum[,vars], group = df.sum$active.t3.miss)

out <- data.frame(do.call("rbind",out))

out <- round(out,2)

out$var <- rownames(out)

out$Group <- str_sub(out$var,end=3)

out$Group <- car::recode(out$Group,"'0_0'='WL MI';'0_1'='WL Obs';'1_0'='Act MI';'1_1'='Act Obs'")

out$Time <- 2

out[grep("t1",out$var),"Time"] <- 1

out$MI <- str_sub(out$var,start=8)

out$MI <- as.numeric(ifelse(out$MI=="",yes="0",no=out$MI))

out$MI <- car::recode(out$MI,"0='MAR';2='0.20';

5='0.50';8='0.80';110='1.10';

140='1.40'")

out$MI <- factor(out$MI,levels=c("MAR","0.20","0.50","0.80","1.10",

"1.40"),ordered=TRUE)

out$Active <- str_sub(out$var,start=1,end=1)

out$Miss <- str_sub(out$var,start=3,end=3)

ggplot(out,aes(y = mean, x = factor(Time), group = Group)) +

geom_point() + geom_line(aes(linetype=Miss,color=Active)) +

facet_wrap(~MI) + theme_bw() + ylab("Mean Distress") +

scale_x_discrete(name="Time",breaks=c(1,2),labels=c("Pre","Post")) +

scale_linetype_discrete(name="Missing",breaks=c(0,1),labels=c("No","Yes")) +

scale_color_discrete(name="Group",breaks=c(0,1),labels=c("WL","Active"))

#For fixed value replacement approach

#Read in original df.rct file

resid.sd <- sd(resid(lm(t3 ~ t1 + active, df.rct)))

df.rct <- df.rct %>%

mutate(resid = resid(lm(t3 ~ t1, data=df.rct, na.action=na.exclude)),

resid.worst = resid,

resid.20 = resid,

resid.50 = resid,

resid.80 = resid) #model without group for residuals

resid.worst <- with(df.rct,max(resid,na.rm=TRUE))

resid.20 <- with(df.rct,mean(resid,na.rm=TRUE)+.20*resid.sd)

resid.50 <- with(df.rct,mean(resid,na.rm=TRUE)+.50*resid.sd)

resid.80 <- with(df.rct,mean(resid,na.rm=TRUE)+.80*resid.sd)

df.rct <- tidyr::replace_na(df.rct, list(resid.worst=resid.worst,resid.20=resid.20,resid.50=resid.50,resid.80=resid.80))

wilcox.test(resid ~ active, data=df.rct) #p < .001

wilcox.test(resid.worst ~ active, data=df.rct) #p = .080

wilcox.test(resid.20 ~ active, data=df.rct) #p = .004

wilcox.test(resid.50 ~ active, data=df.rct) #p = .052

wilcox.test(resid.80 ~ active, data=df.rct) #p = .320

#Adding in rank for plotting / interpretation

rankNA <- function(x) ifelse(is.na(x),NA,rank(x)) #for resid ranks which has NAs

df.rct <- df.rct %>%

mutate(resid.rank = rankNA(resid),

resid.worst.rank = rank(resid.worst),

resid.20.rank = rank(resid.20),

resid.50.rank = rank(resid.50),

resid.80.rank = rank(resid.80))

#summary table

out <- psych::describeBy(df.rct[,c("resid.rank","resid.worst.rank", "resid.20.rank","resid.50.rank","resid.80.rank")],

group = df.rct$active)

out <- data.frame(do.call("rbind",out))

out <- round(out,2)

out$var <- rownames(out)

out <- out[,c("var","n","mean","sd","se")]

out$Group <- c(rep("Passive",5),rep("Active",5))

out$Model <- rep(c("Comp","Worst","0.20","0.50","0.80"),2)

out$Model <- factor(out$Model,levels=c("Comp","Worst","0.20","0.50","0.80"),

ordered=TRUE)

#flip table order

out <- rbind(out[6:10,],out[1:5,])

#add p-values to table

out[out$Group=="Active" & out$Model=="Comp","p"] <-

round(wilcox.test(resid.rank~active,data=df.rct)$p.value,3)

out[out$Group=="Active" & out$Model=="Worst","p"] <-

round(wilcox.test(resid.worst.rank~active,data=df.rct)$p.value,3)

out[out$Group=="Active" & out$Model=="0.20","p"] <-

round(wilcox.test(resid.20.rank~active,data=df.rct)$p.value,3)

out[out$Group=="Active" & out$Model=="0.50","p"] <-

round(wilcox.test(resid.50.rank~active,data=df.rct)$p.value,3)

out[out$Group=="Active" & out$Model=="0.80","p"] <-

round(wilcox.test(resid.80.rank~active,data=df.rct)$p.value,3)

ggplot(out, aes(y = mean, x= Model)) +

geom_bar(stat="identity",position=position_dodge(.9),color="black",aes(group=Group,fill=Group))+

geom_errorbar(aes(ymin=mean-1.96*se, ymax=mean+1.96*se,group=Group), width=.1,position=position_dodge(.9))+

theme_bw() + ylab("Mean Rank")+coord_cartesian(ylim=c(0,230))+

annotate("text", label = "***", x = 1, y = 107, size = 6, colour = "black")+

annotate("text", label = "**", x = 3, y = 215, size = 6, colour = "black")
