# Supplementary material for: Data Missing Not at Random in Mobile Health Research: Assessment of the Problem and a Case for Sensitivity Analyses
Source: J Med Internet Res. 2021 Jun 15;23(6):e26749. doi: 10.2196/26749 (PMC8277392; doi:10.2196/26749)
Supplement: Multimedia Appendix 2 [file jmir_v23i6e26749_app2.docx]

Multimedia Appendix 2. Model-based approaches for handling missing not at random [MNAR] data

Selection models and pattern-mixture models are the most common modern methods for handling MNAR data [1]. Both methods share the feature of modeling the joint distribution between the observed data and the probability of missingness. We do not provide an extensive discussion of these methods and greater detail is included in many missing data analysis texts (e.g., [1,2]). However, a basic understanding could inform mHealth researchers considering various approaches for evaluating the impact of potential MNAR data.

The selection model is a two-part model. The first part is a substantive regression model (e.g., change in distress predicted by group status) and the second part is a regression model predicting the probability of missingness [1]. In theory, simultaneously modeling the two regression equations allows a less biased imputation of missing values than that provided by MAR methods. However, it does come at the expense of assuming missingness mechanisms can be represented through the selection model. When assumptions of the selection model are met, they do successfully reduce MNAR-related bias (e.g., [3]). But these models are highly sensitive and their accuracy can be negatively influenced by mis-specification, collinearity between variables in the substantive and missingness regression models, and the bivariate normality assumption for model residuals [1].

Pattern-mixture models also involve modeling patterns of missingness. For pattern-mixture models, this is done by examining subgroups with shared missing data patterns. Substantive models (e.g., change in distress predicted by group status) are constructed separately within each missingness pattern with results then averaged across models [1]. The nature of missing data typically results in model under-identification, which requires that assumptions be made about some model parameters. For example, the complete case missing variable restriction uses parameters from the complete case analysis to replace parameters that cannot be estimated for a particular missing data pattern. Like selection models, pattern-mixture models can be a powerful means to evaluate bias due to MNAR. However, also like selection models, pattern-mixture models require assumptions, such as the values of parameters that cannot be estimated. Pattern mixture models may be especially relevant for discrete longitudinal applications with multiple measurement time points but can also be applied with just two time points as demonstrated.

**References**

1. Enders CK. Applied Missing Data Analysis. Guilford Press; 2010. ISBN:978-1-60623-639-0

2. Allison PD. Missing Data. SAGE Publications; 2001. ISBN:978-1-4522-0790-2

3. Manning WG, Duan N, Rogers WH. Monte Carlo evidence on the choice between sample selection and two-part models. J Econom 1987 May 1;35(1):59–82. [doi: 10.1016/0304-4076(87)90081-9]
